# 4-Bromo­methyl-6-meth­oxy-2*H*-chromen-2-one

**DOI:** 10.1107/S1600536810042005

**Published:** 2010-10-23

**Authors:** Ramakrishna Gowda, Mahantesha Basanagouda, Manohar V. Kulkarni, K.V. Arjuna Gowda

**Affiliations:** a Department of Physics, Govt. College for Women, Kolar 563 101, Karnataka, India; b Department of Chemistry, Karnatak University, Dharwad 580 003, Karnataka, India; c Department of Physics, Govt. First Grade College, K.R. Pura, Bangalore 560 036, Karnataka, India

## Abstract

The structure of the title coumarin derivative, C_11_H_9_BrO_3_, is stabilized by weak inter­molecular C—H⋯O hydrogen bonds.

## Related literature

For the properties of coumarins, see: Kulkarni *et al.* (2006 ▶); Fylaktakidou *et al.* (2004 ▶); Neyts *et al.* (2009 ▶); Kempen *et al.* (2003 ▶). For structural analysis of coumarins, see: Gnanaguru *et al.* (1985 ▶); Munshi & Guru Row (2005 ▶); Gavuzzo *et al.* (1974 ▶); Moorthy *et al.* (2003 ▶); Katerinopoulos (2004 ▶). For Br-containing coumarins, see: Gaultier & Hauw (1965 ▶); Kokila *et al.* (1996 ▶); Vasudevan *et al.* (1991 ▶).
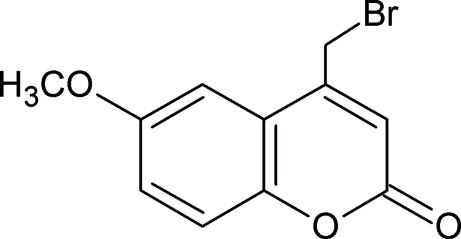


## Experimental

### 

#### Crystal data

C_11_H_9_BrO_3_
*M* *_r_* = 269.09
Monoclinic, 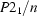

*a* = 4.3573 (3) Å
*b* = 9.2859 (6) Å
*c* = 25.2677 (17) Å
β = 91.927 (3)°
*V* = 1021.79 (12) Å^3^
*Z* = 4
Mo *K*α radiation
μ = 4.01 mm^−1^
*T* = 293 K
0.25 × 0.15 × 0.1 mm

#### Data collection

Bruker Kappa APEXII CCD diffractometer
Absorption correction: multi-scan (*SADABS*; Bruker, 2004 ▶) *T*_min_ = 0.434, *T*_max_ = 0.501
9950 measured reflections
2128 independent reflections
1501 reflections with *I* > 2σ(*I*)
*R*_int_ = 0.037

#### Refinement

*R*[*F* ^2^ > 2σ(*F* ^2^)] = 0.044
*wR*(*F* ^2^) = 0.101
*S* = 0.96
2128 reflections
137 parameters
H-atom parameters constrained
Δρ_max_ = 0.93 e Å^−3^
Δρ_min_ = −0.26 e Å^−3^

### 

Data collection: *APEX2* (Bruker, 2004 ▶); cell refinement: *APEX2* and *SAINT* (Bruker, 2004 ▶); data reduction: *SAINT* (Bruker, 2004 ▶); program(s) used to solve structure: *SIR92* (Altomare *et al.*, 1994 ▶); program(s) used to refine structure: *SHELXL97* (Sheldrick, 2008 ▶); molecular graphics: *ORTEP-3* (Farrugia, 1997 ▶) and *Mercury* (Macrae *et al.*, 2006 ▶); software used to prepare material for publication: *SHELXL97* (Sheldrick, 2008 ▶).

## Supplementary Material

Crystal structure: contains datablocks global, I. DOI: 10.1107/S1600536810042005/bh2314sup1.cif

Structure factors: contains datablocks I. DOI: 10.1107/S1600536810042005/bh2314Isup2.hkl

Additional supplementary materials:  crystallographic information; 3D view; checkCIF report

## Figures and Tables

**Table 1:** Hydrogen-bond geometry (Å, °)

*D*—H⋯*A*	*D*—H	H⋯*A*	*D*⋯*A*	*D*—H⋯*A*
C2—H2⋯O1^i^	0.93	2.60	3.451 (4)	152
C2—H2⋯O2^i^	0.93	2.58	3.446 (5)	155
C10—H10*A*⋯O2^i^	0.97	2.57	3.437 (5)	148
C8—H8⋯O2^ii^	0.93	2.56	3.433 (5)	156
C10—H10*A*⋯O1^iii^	0.97	2.98	3.601 (4)	122

Symmetry codes: (i) 
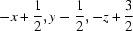
; (ii) 
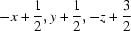
; (iii) 
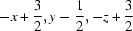
.

**Fig. 1.**
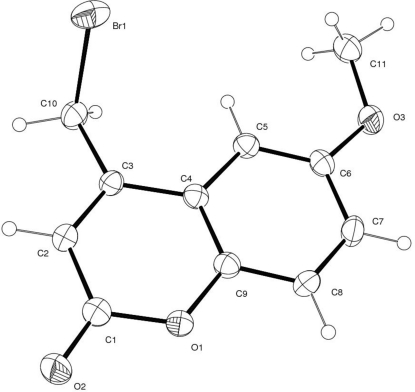
ORTEP diagram of the title molecule with 50% probability displacement ellipsoids for non-H atoms.

## supplementary crystallographic information

### Comment

Coumarins are a class of naturally occurring oxygen heterocycles which have been found to exhibit wide ranging biological activities (Kulkarni *et al.*, 2006; Fylaktakidou *et al.*, 2004; Neyts *et al.*, 2009) through its innumerable derivatives. Structural studies on coumarins have been focused on their solid state photochemical dimerization (Gnanaguru *et al.*, 1985), hydrogen bonding (Munshi *et al.*, 2005), mode of packing (Gavuzzo *et al.*, 1974), molecular self assembling (Moorthy *et al.*, 2003) and photophysical properties (Katerinopoulos *et al.*, 2004). Introduction of bromine has resulted in formation of hydrates, intermolecular hydrogen bonds, and eclipsed conformation, as observed in 3-bromocoumarin (Gaultier *et al.*, 1965), 6-bromo-3-acetylcoumarin (Kokila *et al.*, 1996), and 3-bromoacetylcoumarin (Vasudevan *et al.*, 1991), respectively. 3-Bromophenyl-6-acetoxymethyl-coumarin-3-carboxylates have been found to exhibit potential anticancer and antitumour activity (Kempen *et al.*, 2003).

The title compound is cyclic, planar and aromatic in nature due to the continuous delocalization of electrons over the coumarin rings system. There is a significant deviation from trigonality in bond angle at O1—C1—C2 [117.0 (3)°], due to the electronic repulsion of atom O2 which is bonded to C1. This is also reflected at C9—C4—C5 [117.9 (3)°] and C9—C4—C3 [117.6 (3)°] but these are due to fused benzene and α pyrone rings. Another significant deviation in bond angle is observed at C6—O3—C11 [118.0 (3)°] due to the repulsion between lone pair electrons of atom O3 with valence electrons of C6—O3 and O3—C11 bonds.

### Experimental

To a mixture of equimolar quantity of 4-methoxy phenol (0.1 mol) and 4-bromoethylacetoacetate (0.1 mol) was added drop wise sulfuric acid (30 ml) with stirring and maintaining the temperature between 0-5 °C. The reaction mixture was allowed to stand in ice chest overnight and deep red coloured solution was poured into the stream of crushed ice. Solid separated was filtered and washed with water and then with cold ethanol so as to get a colourless compound. Finally, it is recrystallized from acetic acid.

### Refinement

All the H atoms were positioned geometrically and refined using a riding model with bond lengths 0.97 (methylene), 0.96 (methyl) or 0.93 Å (aromatic). Isotropic displacement parameters were calculated as *U*ĩso~(H) = 1.5*U*~eq~(C) for methyl group C11 and *U*ĩso~(H) = 1.2*U*~eq~(C) for all other H atoms.

### Crystal data

**Table:**

C_11_H_9_BrO_3_	*F*(000) = 536
*M**_r_* = 269.09	*D*_x_ = 1.749 Mg m^−^^3^
Monoclinic, *P*2_1_/*n*	Mo *K*α radiation, λ = 0.71073 Å
Hall symbol: -P 2yn	Cell parameters from 3217 reflections
*a* = 4.3573 (3) Å	θ = 2.3–25.4°
*b* = 9.2859 (6) Å	µ = 4.01 mm^−^^1^
*c* = 25.2677 (17) Å	*T* = 293 K
β = 91.927 (3)°	Needle, colourless
*V* = 1021.79 (12) Å^3^	0.25 × 0.15 × 0.1 mm
*Z* = 4	

### Data collection

**Table:**

Bruker Kappa APEXII CCD diffractometer	2128 independent reflections
Radiation source: fine-focus sealed tube	1501 reflections with *I* > 2σ(*I*)
graphite	*R*_int_ = 0.037
ω and φ scans	θ_max_ = 26.6°, θ_min_ = 2.3°
Absorption correction: multi-scan (*SADABS*; Bruker, 2004)	*h* = −5→3
*T*_min_ = 0.434, *T*_max_ = 0.501	*k* = −11→11
9950 measured reflections	*l* = −31→31

### Refinement

**Table:**

Refinement on *F*^2^	Primary atom site location: structure-invariant direct methods
Least-squares matrix: full	Secondary atom site location: difference Fourier map
*R*[*F*^2^ > 2σ(*F*^2^)] = 0.044	Hydrogen site location: inferred from neighbouring sites
*wR*(*F*^2^) = 0.101	H-atom parameters constrained
*S* = 0.96	*w* = 1/[σ^2^(*F*_o_^2^) + (0.0467*P*)^2^ + 1.203*P*] where *P* = (*F*_o_^2^ + 2*F*_c_^2^)/3
2128 reflections	(Δ/σ)_max_ = 0.008
137 parameters	Δρ_max_ = 0.93 e Å^−^^3^
0 restraints	Δρ_min_ = −0.26 e Å^−^^3^
0 constraints	

### Fractional atomic coordinates and isotropic or equivalent isotropic displacement parameters (Å^2^)

**Table:**

	*x*	*y*	*z*	*U*_iso_*/*U*_eq_	
C1	0.3658 (8)	0.2636 (4)	0.73840 (14)	0.0406 (9)	
C2	0.4641 (8)	0.1339 (4)	0.71330 (13)	0.0375 (8)	
H2	0.3944	0.0462	0.7260	0.045*	
C3	0.6518 (7)	0.1332 (4)	0.67239 (13)	0.0321 (7)	
C4	0.7577 (7)	0.2706 (4)	0.65171 (12)	0.0318 (7)	
C5	0.9489 (7)	0.2851 (4)	0.60828 (13)	0.0348 (8)	
H5	1.0139	0.2035	0.5905	0.042*	
C6	1.0401 (7)	0.4188 (4)	0.59198 (13)	0.0378 (9)	
C7	0.9456 (8)	0.5407 (4)	0.61872 (14)	0.0429 (9)	
H7	1.0121	0.6311	0.6081	0.051*	
C8	0.7556 (8)	0.5293 (4)	0.66048 (14)	0.0401 (9)	
H8	0.6889	0.6113	0.6778	0.048*	
C9	0.6642 (7)	0.3941 (4)	0.67652 (13)	0.0345 (8)	
C10	0.7530 (9)	−0.0074 (4)	0.65024 (14)	0.0421 (9)	
H10A	0.7086	−0.0837	0.6751	0.050*	
H10B	0.9733	−0.0052	0.6460	0.050*	
C11	1.2815 (9)	0.3270 (5)	0.51587 (15)	0.0535 (11)	
H11A	1.4005	0.2554	0.5347	0.080*	
H11B	1.3939	0.3612	0.4863	0.080*	
H11C	1.0908	0.2856	0.5033	0.080*	
O1	0.4744 (5)	0.3905 (3)	0.71899 (9)	0.0397 (6)	
O2	0.1939 (7)	0.2719 (3)	0.77495 (11)	0.0569 (7)	
O3	1.2213 (6)	0.4438 (3)	0.55030 (10)	0.0500 (7)	
Br1	0.54925 (10)	−0.04981 (5)	0.582134 (17)	0.0612 (2)	

### Atomic displacement parameters (Å^2^)

**Table:**

	*U*^11^	*U*^22^	*U*^33^	*U*^12^	*U*^13^	*U*^23^
C1	0.0437 (19)	0.039 (2)	0.039 (2)	−0.0003 (18)	0.0059 (16)	−0.0004 (17)
C2	0.0442 (19)	0.029 (2)	0.0390 (19)	−0.0025 (16)	0.0024 (16)	0.0004 (16)
C3	0.0345 (17)	0.027 (2)	0.0344 (18)	0.0024 (15)	0.0010 (14)	−0.0003 (14)
C4	0.0310 (16)	0.034 (2)	0.0301 (17)	0.0032 (15)	−0.0022 (13)	0.0012 (15)
C5	0.0338 (17)	0.036 (2)	0.0350 (18)	0.0037 (16)	0.0031 (14)	−0.0014 (16)
C6	0.0371 (18)	0.042 (2)	0.0343 (19)	−0.0030 (16)	0.0050 (15)	0.0044 (15)
C7	0.051 (2)	0.030 (2)	0.048 (2)	−0.0055 (18)	0.0027 (17)	0.0076 (17)
C8	0.050 (2)	0.029 (2)	0.042 (2)	0.0018 (17)	0.0008 (16)	−0.0045 (16)
C9	0.0335 (17)	0.037 (2)	0.0333 (18)	−0.0008 (15)	0.0020 (14)	−0.0007 (15)
C10	0.048 (2)	0.032 (2)	0.046 (2)	0.0050 (17)	0.0039 (17)	0.0013 (17)
C11	0.063 (3)	0.053 (3)	0.045 (2)	0.000 (2)	0.0162 (19)	0.002 (2)
O1	0.0483 (14)	0.0339 (15)	0.0376 (13)	0.0013 (12)	0.0117 (11)	−0.0024 (11)
O2	0.0759 (19)	0.0461 (18)	0.0508 (16)	0.0039 (15)	0.0305 (15)	−0.0005 (13)
O3	0.0618 (16)	0.0435 (17)	0.0460 (15)	−0.0072 (13)	0.0208 (13)	0.0030 (13)
Br1	0.0732 (3)	0.0518 (3)	0.0587 (3)	0.0043 (2)	0.0017 (2)	−0.0214 (2)

### Geometric parameters (Å, °)

**Table:**

C1—O2	1.211 (4)	C7—C8	1.367 (5)
C1—O1	1.367 (4)	C7—H7	0.9300
C1—C2	1.434 (5)	C8—C9	1.382 (5)
C2—C3	1.340 (5)	C8—H8	0.9300
C2—H2	0.9300	C9—O1	1.377 (4)
C3—C4	1.459 (5)	C10—Br1	1.950 (4)
C3—C10	1.493 (5)	C10—H10A	0.9700
C4—C9	1.375 (5)	C10—H10B	0.9700
C4—C5	1.406 (5)	C11—O3	1.420 (5)
C5—C6	1.371 (5)	C11—H11A	0.9600
C5—H5	0.9300	C11—H11B	0.9600
C6—O3	1.357 (4)	C11—H11C	0.9600
C6—C7	1.388 (5)		
			
O2—C1—O1	116.7 (3)	C7—C8—C9	119.0 (3)
O2—C1—C2	126.3 (4)	C7—C8—H8	120.5
O1—C1—C2	117.0 (3)	C9—C8—H8	120.5
C3—C2—C1	123.0 (3)	C4—C9—C8	122.1 (3)
C3—C2—H2	118.5	C4—C9—O1	122.0 (3)
C1—C2—H2	118.5	C8—C9—O1	115.9 (3)
C2—C3—C4	118.7 (3)	C3—C10—Br1	112.1 (2)
C2—C3—C10	119.3 (3)	C3—C10—H10A	109.2
C4—C3—C10	122.0 (3)	Br1—C10—H10A	109.2
C9—C4—C5	117.9 (3)	C3—C10—H10B	109.2
C9—C4—C3	117.6 (3)	Br1—C10—H10B	109.2
C5—C4—C3	124.5 (3)	H10A—C10—H10B	107.9
C6—C5—C4	120.4 (3)	O3—C11—H11A	109.5
C6—C5—H5	119.8	O3—C11—H11B	109.5
C4—C5—H5	119.8	H11A—C11—H11B	109.5
O3—C6—C5	124.7 (3)	O3—C11—H11C	109.5
O3—C6—C7	115.4 (3)	H11A—C11—H11C	109.5
C5—C6—C7	119.9 (3)	H11B—C11—H11C	109.5
C8—C7—C6	120.7 (3)	C1—O1—C9	121.6 (3)
C8—C7—H7	119.7	C6—O3—C11	118.0 (3)
C6—C7—H7	119.7		
			
O2—C1—C2—C3	179.0 (3)	C5—C4—C9—C8	−0.9 (5)
O1—C1—C2—C3	−0.5 (5)	C3—C4—C9—C8	179.0 (3)
C1—C2—C3—C4	−1.0 (5)	C5—C4—C9—O1	179.3 (3)
C1—C2—C3—C10	177.4 (3)	C3—C4—C9—O1	−0.8 (4)
C2—C3—C4—C9	1.6 (4)	C7—C8—C9—C4	−0.2 (5)
C10—C3—C4—C9	−176.7 (3)	C7—C8—C9—O1	179.6 (3)
C2—C3—C4—C5	−178.5 (3)	C2—C3—C10—Br1	106.3 (3)
C10—C3—C4—C5	3.2 (5)	C4—C3—C10—Br1	−75.4 (3)
C9—C4—C5—C6	0.7 (5)	O2—C1—O1—C9	−178.1 (3)
C3—C4—C5—C6	−179.2 (3)	C2—C1—O1—C9	1.4 (5)
C4—C5—C6—O3	−179.3 (3)	C4—C9—O1—C1	−0.8 (5)
C4—C5—C6—C7	0.5 (5)	C8—C9—O1—C1	179.4 (3)
O3—C6—C7—C8	178.2 (3)	C5—C6—O3—C11	10.6 (5)
C5—C6—C7—C8	−1.6 (5)	C7—C6—O3—C11	−169.2 (3)
C6—C7—C8—C9	1.4 (5)		

### Hydrogen-bond geometry (Å, °)

**Table:**

*D*—H···*A*	*D*—H	H···*A*	*D*···*A*	*D*—H···*A*
C2—H2···O1^i^	0.93	2.60	3.451 (4)	152
C2—H2···O2^i^	0.93	2.58	3.446 (5)	155
C10—H10A···O2^i^	0.97	2.57	3.437 (5)	148
C8—H8···O2^ii^	0.93	2.56	3.433 (5)	156
C10—H10A···O1^iii^	0.97	2.98	3.601 (4)	122

Symmetry codes: (i) −*x*+1/2, *y*−1/2, −*z*+3/2; (ii) −*x*+1/2, *y*+1/2, −*z*+3/2; (iii) −*x*+3/2, *y*−1/2, −*z*+3/2.
